# A Cross-Sectional Study Comparing Two Opt-Out HIV Testing Strategies in the Out-Patient Setting

**DOI:** 10.3389/fpubh.2021.664494

**Published:** 2021-06-11

**Authors:** Greta Tam, Samuel Yeung Shan Wong

**Affiliations:** ^1^School of Public Health, The University of Hong Kong, Hong Kong, China; ^2^Jockey Club School of Public Health and Primary Care, The Chinese University of Hong Kong, Hong Kong, China

**Keywords:** acceptability, out-patient, testing, HIV, opt-out, opt-out approach

## Abstract

**Background:** HIV infections are generally asymptomatic, leading to undetected infections and late-stage diagnoses. There are a lack of acceptable testing strategies for routine opt-out HIV screening. Our aim was to evaluate and compare the diagnostic yield of routine opt-out HIV testing strategies in two out-patient settings in a low HIV prevalence country: The public primary care and specialist out-patient care setting

**Methods:** A cross-sectional study was conducted in a primary care clinic over a four-week period in 2016 to 2017 and in a specialist out-patient clinic over a concurrent 11-month period. Patients were invited to complete a questionnaire assessing demographic characteristics, acceptance of opt-out HIV testing as a policy in all out-patient clinics in Hong Kong and reasons if refusing the HIV test. All respondents were offered an HIV test.

**Results:** This study included 648 and 1,603 patients in the primary care and specialist out-patient clinic, respectively. Test acceptability was 86 and 87% in the primary care and specialist out-patient setting, respectively. Test uptake was 35 and 68% in the primary care and specialist out-patient setting, respectively. No HIV infections were detected.

**Conclusion:** Opt-out HIV testing during routine blood taking in the specialist out-patient setting achieved a high test uptake and acceptability. In contrast, opt-out HIV testing using rapid finger-prick tests in the primary care setting was not effective.

## Introduction

Human Immunodeficiency Virus (HIV), one of the most serious public health issues worldwide, generally presents as a non-specific flu-like illness followed by an asymptomatic period of a decade or longer, with a quarter of infections remaining undiagnosed (1, 2). With the advent of highly active antiretroviral therapy (HAART), a near normal life expectancy is possible with timely treatment (3). Early diagnosis can also lead to treatment as prevention (TasP) which decreases risk behavior and HIV transmission (4). Strategies to detect HIV in the general population have produced varying results, with acceptance rates at in-patient settings as low as 21–24% and more than half of patients excluded from the study as they were too ill. In addition, fear of venipuncture was a commonly cited reason for refusing HIV test (5, 6).

Using an out-patient setting may overcome some of the barriers to testing experienced in the in-patient setting. Patients will not be too ill to participate in the study and extra venipuncture can be avoided by incorporating HIV testing into routine blood-taking or using finger-prick test. In countries with low HIV prevalence, such as the United Kingdom (0.2%) and Hong Kong (0.1%), screening in specialist sexual health services accounts for most people who are tested for HIV (7–10). In order to detect all cases of HIV infection, not just those in risk groups, a setting most representative of the general population could help to detect cases that are usually hidden. However, a high acceptance rate is important to increase the number of diagnoses and therefore diagnostic yield in a low-prevalence setting. Public healthcare is available to the general population of Hong Kong at minimal cost. Although the patient population is not completely representative of the general human population in Hong Kong, it is the most representative of Hong Kong's general population that is possible in a clinic setting. In primary care, patients usually present for acute problems and minor ailments which have a lower tendency to require further investigations, thus necessitating the use of finger-prick HIV testing to avoid venipuncture. In the specialist out-patient clinic, follow-up of chronic diseases often involves routine blood tests, whereby HIV testing could be incorporated. Our aim was to evaluate and compare the diagnostic yield of routine opt-out HIV testing strategies in two out-patient settings in a low HIV prevalence country: The public primary care and specialist out-patient care setting.

## Methods

### Testing Strategy

**Figure 1:**
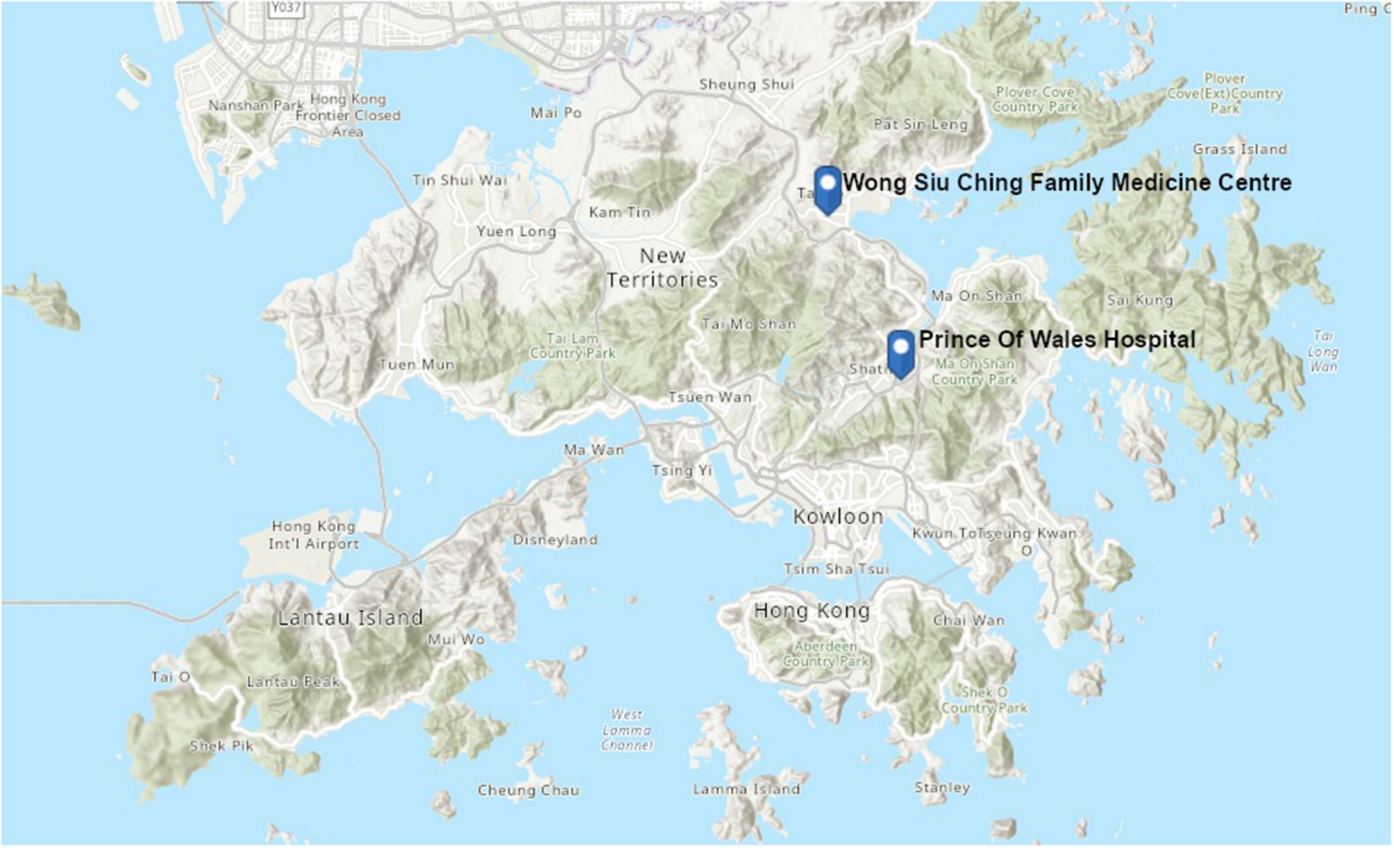
Map showing study area in Hong Kong.

A primary care clinic and hepatology clinic were approached by the principal investigator to take part in the study. The Wong Siu Ching primary care clinic (Figure 1) serves a large population in the Hong Kong community. It is staffed by residents, specialists and consultants. The Prince of Wales Hospital hepatology clinic (Figure 1) provides specialist care to patients with chronic hepatitis B or C and other chronic liver diseases.

Over a 4-week period, 648 patients were approached by non-clinical staff at the waiting area and offered HIV testing at the primary care clinic. Over a concurrent 11-month period, 1,603 patients were also approached by non-clinical staff at the waiting area and offered HIV testing at the hepatology clinic. Patients were given pamphlets explaining the study. Tests were free of charge. Dried blood spot finger-prick test was administered at the primary care clinic, while blood for HIV testing was taken during routine blood-taking at the phlebotomy centre for hepatology patients. All participants regardless of whether they were tested were invited to complete a short questionnaire. Participants provided written informed consent for the study. The study was approved by the university ethics committee (CRE Ref. No. 2015.718_FR).

### Testing Strategy Implementation

Patients above 18 years old are invited to participate in an HIV blood test. Blood samples were transported in the same afternoon or evening that they were collected to the laboratory. Samples repeatedly reactive to the 4th generation HIV-1/2 EIA screening assays (detecting antibody to HIV-1 and 2, plus HIV-1 p24 antigen) were retested with the confirmatory assay GeeniusTMHIV1/2 (Bio-Rad). For positive samples, a western blot was done for confirmation. A serial number was provided, and each tested person advised to call back a telephone number for results after 1 week. Patients who had positive test results were contacted by a physician then counseled by staff of the non-governmental organization AIDS concern and referred to the Department of Health's HIV clinic for follow-up.

### Data Collection

Questionnaires included the following: age, gender, place of birth, whether participants agreed with the policy of providing HIV screening as an opt-out test at all primary care and specialist outpatient clinics. Those who opted out of HIV testing were also asked their reasons for refusing.

### Statistical Analysis

The testing strategy was evaluated by HIV test acceptance rates and positive test results. HIV test acceptance rates were evaluated by patient acceptability from questionnaires and uptake of HIV testing. We expected a test uptake of 60% in the primary care setting and 70% in the specialist out-patient setting based on previous studies (11, 12). A rate of positive test results higher than 0.1% would be considered promising, as this was the threshold deemed as cost-effective (11, 12). Rates of test uptake, patient acceptability from questionnaires and positive test results were calculated. Chi-square test was used to assess socio-demographic factors associated with refusal. Analyses were performed using SPSS 25.0 (IBM Inc).

This study was approved by the Survey and Behavioural Research Ethics Committee of The Chinese University of Hong Kong. Written consent was obtained from each of the participants at the beginning of the study.

## Results

### Test Acceptability From Questionnaires

At the primary care clinic, 70% of approached patients agreed to participate in the study (456/648), while at the specialist out-patient clinic 89% participated (1421/1603). All study participants answered the questionnaires, regardless whether they opted out of HIV testing. 86% (394/456) of study participants at the primary care clinic agreed with the policy of providing opt-out HIV testing in all public primary care and specialist out-patient clinic settings. A similar proportion of study participants at the specialist out-patient clinic agreed with this policy: 87% (1231/1421). Among study participants at the primary care clinic, 38% were male, while at the specialist out-patient clinic the gender ratio was reversed (38% were female). The median age of participants at the primary care clinic was 55 years (IQR = 41–61 years), with a similar median at the specialist out-patient clinic of 54 years (IQR = 43–61.5). At the primary care clinic, 65% of participants were born in Hong Kong and 33% were born in Mainland China. At the specialist out-patient clinic, 58% of participants were born in Hong Kong and 40% were born in Mainland China.

### Test Uptake and Rate of Positive Test Results

At the primary care clinic, test uptake was 35% (*n* = 160) compared to 68% (*n* = 966) at the specialist out-patient clinic. The rate of positive HIV test results was 0% overall.

### Determinants of Test Acceptability and Uptake

Test uptake was higher among men (66%) and those born in Mainland China (36%) in the specialist out-patient clinic compared to the primary care clinic. There was no difference in age of participants accepting test and test uptake between the two out-patient settings (Table 1).

**Table 1:** Sociodemographic determinants of accepting test and test uptake.

**Determinant**	**Primary care clinic % (number of sample size)**	**Specialist out-patient clinic % (number of sample size)**	***P*-value**	**Chi-square value**
Male gender	44 (67)	66 (596)	0.00000037	25.84
Age (median, quartiles)	53 (38–60)	52 (42–60)	0.63	0.23
**Place of birth:**				
Hong Kong	70 (106)	62 (564)	0.06	3.42
Mainland China	28 (42)	36 (330)	0.07	3.28

At the primary care clinic, the main reasons for opting out of testing were perceived low risk of infection (64%, *n* = 292) and fear of venipuncture (16%, *n* = 73) (Table 2). Perceived low risk of infection was also the top reason for opting out of testing at the specialist out-patient clinic (57%, *n* = 810). However, the other main reason was having taken a lot of blood already (14%, *n* = 199). Reasons based on fear of stigma was rare in both clinics (1%, *n* = 5 in primary care clinic and 3%, *n* = 43 in specialist out-patient clinic).

**Table 2:** Reasons for opting out of testing.

**Reasons**	**Primary care clinic % (number of sample size)**	**Specialist out-patient clinic % (number of sample size)**
Perceived low risk of infection	64 (292)	57 (810)
Fear of venipuncture	16 (73)	4 (57)
Tested before	5 (23)	9 (128)
Do not want to know test result	4 (18)	5 (71)
Taken a lot of blood	2 (9)	14 (199)
Fear of stigma	1 (5)	3 (43)
Other	8 (36)	8 (113)

## Discussion

An opt-out testing strategy to detect hidden HIV infections in both the general population and high-risk population had a limited diagnostic yield in terms of rate of positive results. Despite failing to detect hidden HIV infections, this strategy had high acceptability in both settings. Meanwhile test uptake was lower than expected in the primary care setting and similar to the expected rate in the specialist out-patient setting.

Test uptake of 35% in the primary care setting was lower than that of international studies which offered opt-out rapid HIV testing in a similar setting (59–62%). The low uptake could be due to the lack of healthcare personnel offering the test: The test was offered by research assistants in our study whereas HIV testing was offered by clinicians and healthcare assistants in the international studies (11). A previous study concluded that the primary care doctor's personal invitation was key to a high uptake rate as this was the main reason cited by patients to get tested (13). Test uptake of 68% in the specialist out-patient setting was higher than in the primary care setting and comparable to international studies where HIV testing was offered at genitourinary medicine clinics (12). The high uptake could be due to the relative ease of testing: In the specialist clinic setting, HIV testing was performed within the routine diagnostic work-up.

Regardless of whether the setting was within a low prevalence (primary care) or high-risk (hepatology clinic) population, there was a low perceived risk of infection—the main barrier to testing. A similar phenomenon is seen worldwide (6, 14–17). Methods for HIV screening remained a problem and was the second most commonly cited reason for opting out; In the primary care setting, use of rapid finger-prick testing failed to ease fears of venipuncture. Although in the specialist clinic setting these fears were bypassed, patients felt that a lot of blood had already been taken during the routine diagnostic work-up. This could be specific to our study setting in a university hospital where other studies were concurrently running, offering additional blood tests. In addition, although blood was taken during routine testing, patients were given additional tubes which they needed to bring to their next blood taking in order to be screened for HIV. This additional step may have felt too bothersome to patients. This barrier could be overcome if the test were incorporated into the health care system.

Our study is the first to directly compare two different HIV opt-out testing strategies, in different populations and using different methods. Our study has some limitations. First, selection bias may be present due to convenience sampling and possible consent bias. Second, the sample size was too small to detect hidden HIV infections. Third, our study was not fully integrated into the healthcare system. In order for the study to be feasible, it was run in parallel to usual care to avoid placing excess burden on healthcare staff. Thus, we were not able to do HIV testing in a truly opt-out manner, whereby the patient was tested unless they declined. Research assistants approached patients, explained the nature of the study and gained consent for participation. The ensuing test offer approximated that of “active choice,” whereby the patient was asked whether they would like to be tested for HIV. Participants were given test tubes to bring to their next routine blood test at the hepatology clinic. There was a separate hotline that patients called for their HIV test results. Active choice testing has been shown to have lower uptake rates compared to truly opt-out testing (18). Underestimation of uptake rates may explain the difference between the high test acceptability rates compared to the lower uptake rates found in our study.

## Conclusions

The testing strategy of opt-out HIV testing during routine blood taking in the specialist out-patient setting was effective in achieving a high test uptake. The high test acceptability rates also suggest that this strategy could be rolled out on a larger scale. The diagnostic yield in terms of positive test results would potentially increase. In contrast, opt-out HIV testing using rapid finger-prick tests in the primary care setting was not effective and is not recommended for countries with a low HIV prevalence.

## Data Availability Statement

The raw data supporting the conclusions of this article will be made available by the authors, without undue reservation.

## Ethics Statement

The studies involving human participants were reviewed and approved by Survey and Behavioural Research Ethics Committee of The Chinese University of Hong Kong. The patients/participants provided their written informed consent to participate in this study.

## Author Contributions

GT participated in planning the study, data collection, interpreting the results, obtained research funding, and wrote the first draft of the article. GT and SW participated in data collection, interpreting the results, and final paper writing. All authors were involved in planning the article, critical review and editing of the first draft, and subsequent revisions to the paper. All authors read and approved the final manuscript.

## Conflict of Interest

The authors declare that the research was conducted in the absence of any commercial or financial relationships that could be construed as a potential conflict of interest.
